# Time-Course Study of the Antibacterial Activity of an Amorphous SiO_x_C_y_H_z_ Coating Certified for Food Contact

**DOI:** 10.3390/antibiotics10080901

**Published:** 2021-07-23

**Authors:** Alessandro Di Cerbo, Giuseppe Rosace, Stefano Rea, Roberta Stocchi, Julio Cesar Morales-Medina, Roberto Canton, Andrea Mescola, Carla Condò, Anna Rita Loschi, Carla Sabia

**Affiliations:** 1 School of Biosciences and Veterinary Medicine, University of Camerino, 62024 Matelica, Italy; stefano.rea@unicam.it (S.R.); roberta.stocchi@unicam.it (R.S.); annarita.loschi@unicam.it (A.R.L.); 2 Department of Engineering and Applied Sciences, University of Bergamo, 24044 Dalmine, Italy; giuseppe.rosace@unibg.it; 3 Centro de Investigación en Reproducción Animal, CINVESTAV-Universidad Autónoma de Tlaxcala, AP 62, Tlaxcala 90000, Mexico; jmoralesm@cinvestav.mx; 4 Moma Nanotech Srl, 20861 Brugherio, Italy; canton@nanotech.it; 5 CNR-Nanoscience Institute-S3, 62024 Modena, Italy; andrea.mescola@nano.cnr.it; 6 Department of Life Sciences, University of Modena and Reggio Emilia, 41125 Modena, Italy; Carla.condo@hotmail.it (C.C.); carla.sabia@unimore.it (C.S.)

**Keywords:** food contact material, stainless steel, SiO_x_C_y_H_z_ coating, hydrophobicity, antibacterial activity

## Abstract

One of the most-used food contact materials is stainless steel (AISI 304L or AISI 316L), owing to its high mechanical strength, cleanability, and corrosion resistance. However, due to the presence of minimal crevices, stainless-steel is subject to microbial contamination with consequent significant reverb on health and industry costs due to the lack of effective reliability of sanitizing agents and procedures. In this study, we evaluated the noncytotoxic effect of an amorphous SiO_x_C_y_H_z_ coating deposited on stainless-steel disks and performed a time-course evaluation for four Gram-negative bacteria and four Gram-positive bacteria. A low cytotoxicity of the SiO_x_C_y_H_z_ coating was observed; moreover, except for some samples, a five-logarithm decrease was visible after 1 h on coated surfaces without any sanitizing treatment and inoculated with Gram-negative and Gram-positive bacteria. Conversely, a complete bacterial removal was observed after 30 s^−1^ min application of alcohol and already after 15 s under UVC irradiation against both bacterial groups. Moreover, coating deposition changed the wetting behaviors of treated samples, with contact angles increasing from 90.25° to 113.73°, realizing a transformation from hydrophilicity to hydrophobicity, with tremendous repercussions in various technological applications, including the food industry.

## 1. Introduction

Among food contact materials (FCM), metals and alloys (Fe, Ni, Cr, and/or Mo) are the most widely employed in the food industry for equipment (pasteurizers, separators, pipework) and utensils (knives, salami tongs, meat tenderizers, forks, skimmers) [1].

When selecting metals or alloys to produce equipment and utensils, it is necessary to take into account several aspects, such as operative pH and temperature values, maintenance requirements, hygienic properties, safety, organoleptic inertness, and the food it will come into contact with. In this sense, general requirements for FCM are reported in the Regulation (EC) No. 1935/2004, while good manufacturing practices for their production are included in the Regulation (EC) No. 2023/2006 [2,3].

In addition, according to Regulation (EC) No. 333/2007, FCM share the feature of releasing as few substances (e.g., metal ions) as possible in order to not harm human health or to not induce changes in the food composition that may cause spoilage [4].

However, due to the presence of minimal crevices, FCM are also subject to microbial contamination and, consequently, become a potential source of food contamination as well [5].

Therefore, an accurate selection and correct use of disinfectants such as alcohol, UVC radiation, iodine, biguanide, quaternary ammonium compounds, peracetic acid, and sodium hypochlorite are fundamental to prevent cross contamination or antimicrobial resistance on FCM [6,7].

One of the most-used FCM is stainless steel (AISI 304L or AISI 316L), owing to its high mechanical strength, cleanability, and corrosion resistance [5,8]. Nevertheless, hydrophobicity, contact angle, hydration, charge, surface chemistry, roughness (expressed as an average surface roughness (*R_a_*) and/or root-mean-square surface roughness (*R*_rms_)), and pore presence also play a pivotal role in terms of bacterial adhesion and sanitizing procedure outcome [9,10,11,12,13,14].

For instance, it has been observed that surface chemistry alteration through the grafting of functional groups changed the surface wettability, thus hindering bacterial adhesion [15,16]. Furthermore, industrial food-processing surfaces with a roughness higher than 0.8 μm were proven to enhance bacterial adhesion due to the presence of depressions able to protect bacteria from shear forces, environmental disturbances, and sanitizing procedures [17]. At the same time, the overcoming of the surface energy barrier by the bacteria through electrostatic repulsion can also provide favorable conditions for bacterial adhesion [18].

In addition, contact angle can influence microbial contamination, as low values have been strongly correlated with an antibacterial behavior of the surface [19].

The most common causes of food-processing plant contamination are pathogenic and/or spoilage bacteria, such as *Listeria monocytogenes*, *Staphylococcus aureus*, *Bacillus cereus*, *Salmonella enterica*, *Campylobacter* spp., enterohemorrhagic *Escherichia coli*, *Shigella* spp., *Vibrio* spp., *Pseudomonas* spp., *Acinetobacter* spp., *Moraxella* spp., *Brochothrix thermosphacta*, *Shewanella putrefaciens*, *Lactobacillus* spp., *Leuconostoc* spp., and *Enterococcus* spp., many of which are able to produce biofilms [20], with consequent significant reverb on health and industry costs due to the lack of effective reliability of sanitizing agents and procedures [21,22].

In order to overcome organic fouling and microbial colonization of stainless-steel surfaces, several strategies, e.g., H^+^, F^+^, and Si^+^ implantation [23] or SiO_x_, poly(ethylene glycol) [24], poly(tetrafluoroethylene), and electroless nickel-PTFE [25] coating have been proposed.

In this study, we firstly aimed to confirm the antibacterial and noncytotoxic effect of an amorphous SiO_x_C_y_H_z_ coating, named Nanoxham-D^®^, deposited on stainless-steel disks. Then, we also performed a time-course evaluation to better address the time required to achieve the lowest bacterial concentration and provide further insights into the reliability of two different sanitizing agents applied on the coating itself.

## 2. Results

### 2.1. Time-Course Assay

**Figure 1:**
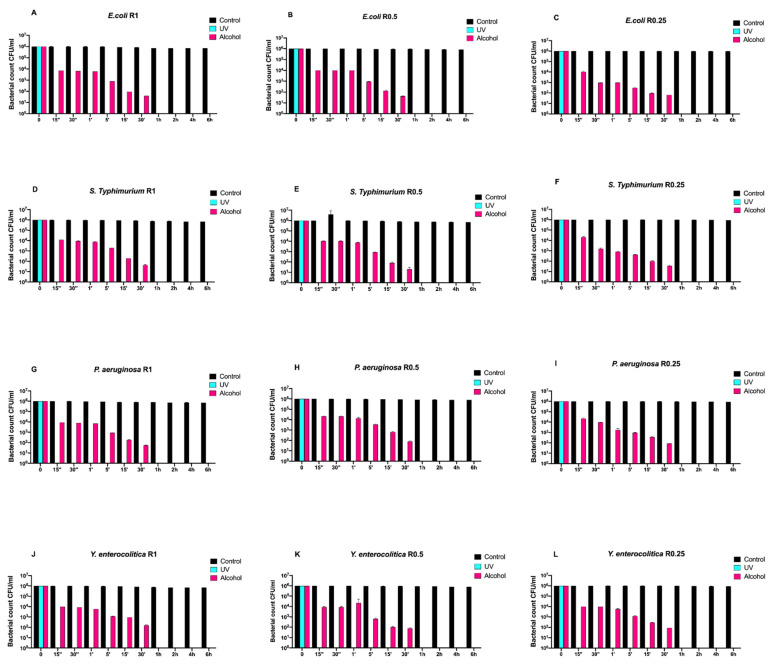
Graphical representation of the different exposure times of uncoated disks with different surface roughness (R0.25, R0.5, and R1) unsanitized (control) and subjected to two sanitizing methods (UVC and alcohol) against (**A**–**C**) *Escherichia coli* ATCC 25922, (**D**–**F**) *Salmonella Typhimurium* ATCC 1402, (**G**–**I**) *Pseudomonas aeruginosa* ATCC 27588 and (**J**–**L**) *Yersinia enterocolitica* ATCC 9610.

Figure 1 reports the Gram-negative (*Escherichia coli* ATCC 25922, *Salmonella Typhimurium* ATCC 1402, *Yersinia enterocolitica* ATCC 9610, and *Pseudomonas aeruginosa* ATCC 27588) count (vertical axes) as a function of the exposure time (abscissa) for different surface roughnesses (the three columns refer to R0.25, R0.5, and R1, respectively). Black, turquoise, and purple bars refer to control (untreated with disinfectant), UVC-treated, and alcohol-treated, respectively.

Regardless of roughness, all control samples did not induce any bacterial count decrease, while alcohol treatment completely eliminated all bacteria after 30 min.

Conversely, the complete elimination of bacteria was achieved by means of UV treatment already after 15 s of exposure (Figure 1A–L).

**Figure 2:**
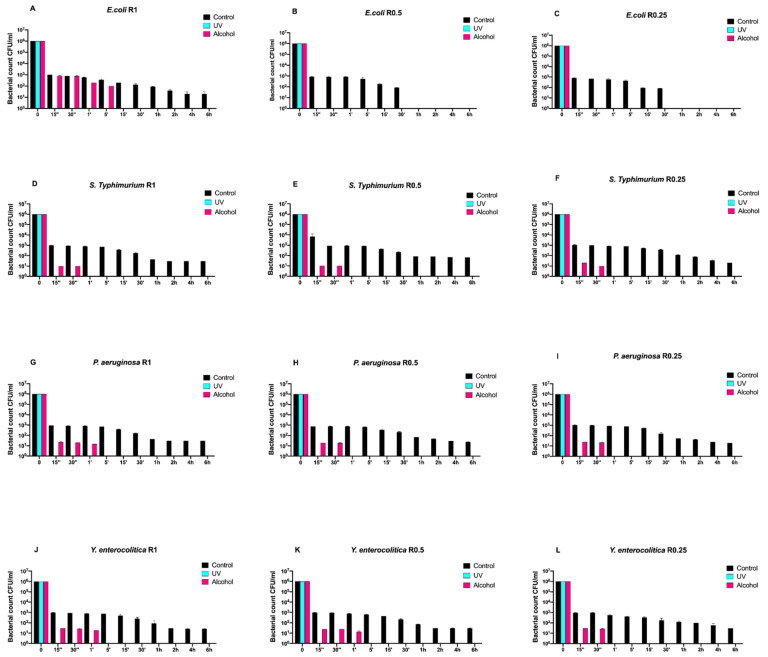
Graphical representation of the different exposure times of SiO_x_C_y_H_z_-coated disks with different surface roughness (R0.25, R0.5, and R1) unsanitized (control) and subjected to two sanitizing methods (UVC and alcohol) against (**A**–**C**) *Escherichia coli* ATCC 25922, (**D**–**F**) *Salmonella Typhimurium* ATCC 1402, (**G**–**I**) *Pseudomonas aeruginosa* ATCC 27588 and (**J**–**L**) *Yersinia enterocolitica* ATCC 9610.

Then, we also evaluated the time-course growth of the same bacteria on SiO_x_C_y_H_z_-coated disks (Figure 2) untreated (control) and treated with the same sanitizing methods (UVC and alcohol).

Interestingly, a three-logarithm decrease, from 10^6^ to 10^3^, was constantly observed for each strain after 15 s in control disks regardless of roughness. A further decrease, from 10^3^ to 10^1^, was also observed for all strains and roughness after 1 h, except for *Escherichia coli* ATCC 25922, which showed a complete removal after 30 min (Figure 2A–L). Moreover, this strain showed a different growth trend after alcohol treatment. In fact, complete removal was achieved after 5 min for R1 and already after 15 s for R0.5 and R0.25 (Figure 2A–C).

As far as it concerns *Salmonella Typhimurium* ATCC 1402, alcohol treatment removed it completely after 30 s regardless of roughness (Figure 2D–F). A similar trend was also observed for *Yersinia enterocolitica* ATCC 9610 for R0.25 and for *Pseudomonas aeruginosa* ATCC 27588 for R0.5 and R0.25, respectively (Figure 2H,I,L).

On the other hand, alcohol treatment achieved a similar result after 1 min for *Pseudomonas aeruginosa* ATCC 27588 for R1 and for *Yersinia enterocolitica* ATCC 9610 for R1 and R0.5 (Figure 2G,J,K), respectively.

**Figure 3:**
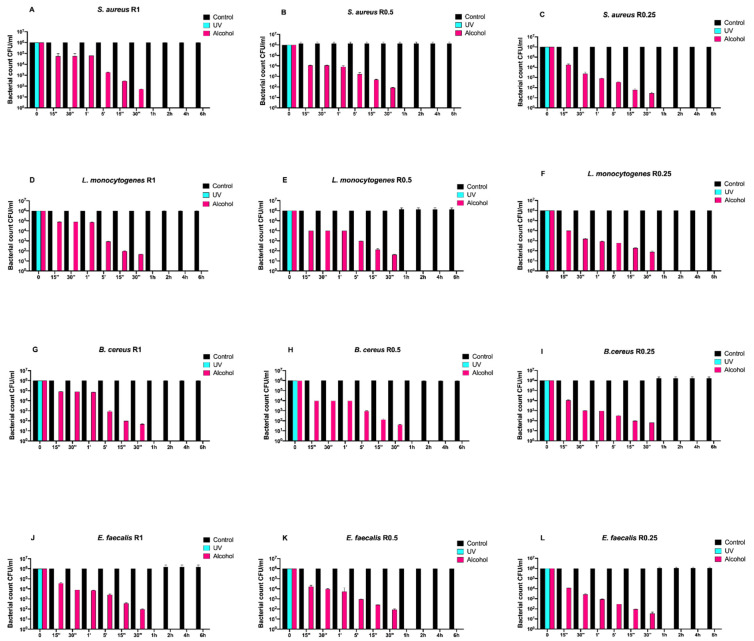
Graphical representation of the different exposure times of uncoated disks with different surface roughness (R0.25, R0.5, and R1) unsanitized (control) and subjected to two sanitizing methods (UVC and alcohol) against (**A**–**C**) *Staphylococcus aureus* ATCC 6538, (**D**–**F**) *Listeria monocytogenes* NCTT 10888, (**G**–**I**) *Bacillus cereus* ATCC 14579 and (**J**–**L**) *Enterococcus faecalis* ATCC 29212.

**Figure 4:**
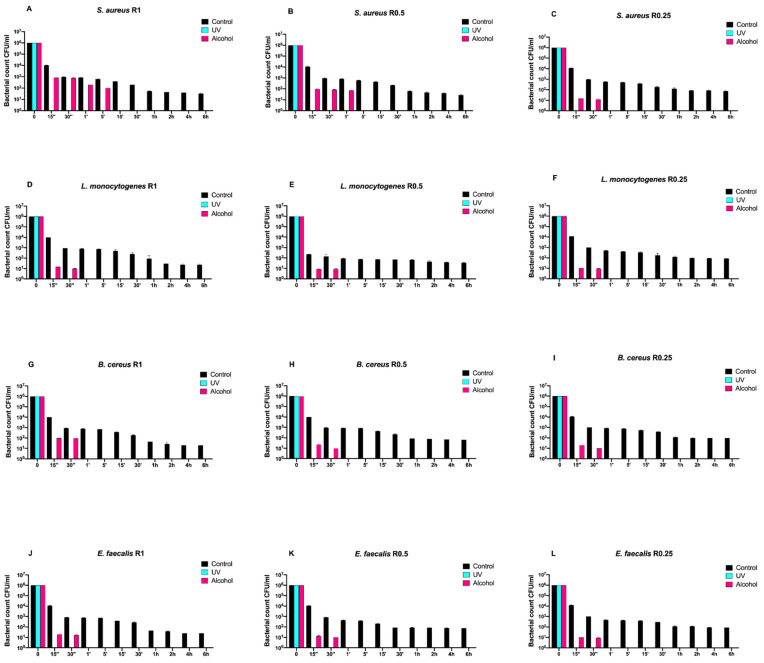
Graphical representation of the different exposure times of SiO_x_C_y_H_z_-coated disks with different surface roughness (R0.25, R0.5, and R1) unsanitized (control) and subjected to two sanitizing methods (UVC and alcohol) against (**A**–**C**) *Staphylococcus aureus* ATCC 6538, (**D**–**F**) *Listeria monocytogenes* NCTT 10888, (**G**–**I**) *Bacillus cereus* ATCC 14579 and (**J**–**L**) *Enterococcus faecalis* ATCC 29212.

Afterwards, we assayed four Gram-positive bacteria (*Staphylococcus aureus* ATCC 6538, *Enterococcus faecalis* ATCC 29212, *Bacillus cereus* ATCC 14579, and *Listeria monocytogenes* NCTT 10888) on both uncoated and SiO_x_C_y_H_z_-coated disks (Figure 3 and Figure 4, respectively).

Regardless of roughness and bacterial strain, the control surface did not induce a decrease in the microbial load, and, at the same time, a five-logarithm decrease from 10^6^ to 10^1^ was generally observed 30 min after alcohol treatment (Figure 3A–L). Conversely, SiO_x_C_y_H_z_-coated disks induced a microbial growth trend similar to that observed for Gram-negative bacteria (Figure 4).

In fact, all controls induced a five-logarithm decrease from 10^6^ to 10^1^ after 6 h. However, *Listeria monocytogenes* NCTT 10888 reached such concentration already after 5 min for R0.5 (Figure 4H), while for *Enterococcus faecalis* ATCC 29212, it was reached after 30 min for R0.5 (Figure 4K). As for *Staphylococcus aureus* ATCC 6538, *Bacillus cereus* ATCC 14579, and *Enterococcus faecalis* ATCC 29212, the 10^1^ logarithm was reached after 1 h for R1 for all the three strains (Figure 4A,G,J) and for R0.5 for the first *Staphylococcus aureus* ATCC 6538 and *Bacillus cereus* ATCC 14579 (Figure 4B,E).

*Staphylococcus aureus* ATCC 6538, *Listeria monocytogenes* NCTT 10888, and *Bacillus cereus* ATCC 14579 reached the 10^1^ logarithm after 2 h for R0.25, R1, and R0.25, respectively (Figure 4C,D,I), while *Enterococcus faecalis* ATCC 29212 and *Listeria monocytogenes* NCTT 10888 reached it for R0.25 after 4 and 6 h, respectively (Figure 4F,L).

Dealing with alcohol treatment, all bacterial strains were completely removed after 30 s for all roughness, except for *Staphylococcus aureus* ATCC 6538, which was successfully removed after 1 and 5 min for R1 and R0.5, respectively (Figure 4A,B).

### 2.2. Neutral Red Assay

**Figure 5:**
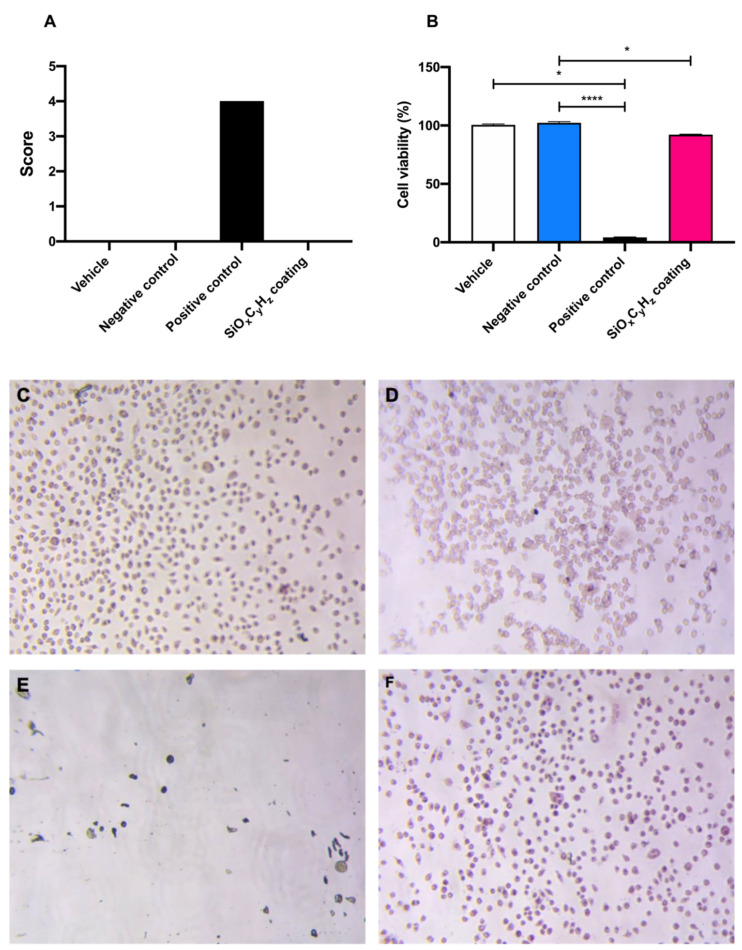
Graphical representation of (**A**) morphological grading score, (**B**) cell viability, and (**C**–**F**) microscopic images (10×) of vehicle, negative and positive control, and SiO_x_C_y_H_z_ coating, respectively; * *p* < 0.05 and **** *p* < 0.0001.

In Figure 5, morphological grading score, cell viability of SiO_x_C_y_H_z_ coating, and controls are reported.

Morphological grading score revealed no differences among high density polyethylene USP reference standard, supplemented culture medium, and SiO_x_C_y_H_z_ coating extracts; on the other hand, latex reached a score of four, inducing almost complete destruction of the cell layer (Figure 5A,C–E). Furthermore, cell viability assay showed a significant cell growth inhibition after the 24 h incubation with both latex (**** *p* < 0.001) and Nanoxham-D (* *p* < 0.05) extracts, with respect to polyethylene USP reference standard and supplemented culture medium (Figure 5B–E). However, since the cell viability reduction induced by Nanoxham-D extract was <30%, it was considered noncytotoxic.

### 2.3. Environmental Scanning Microscopy Analysis (ESEM)

**Figure 6:**
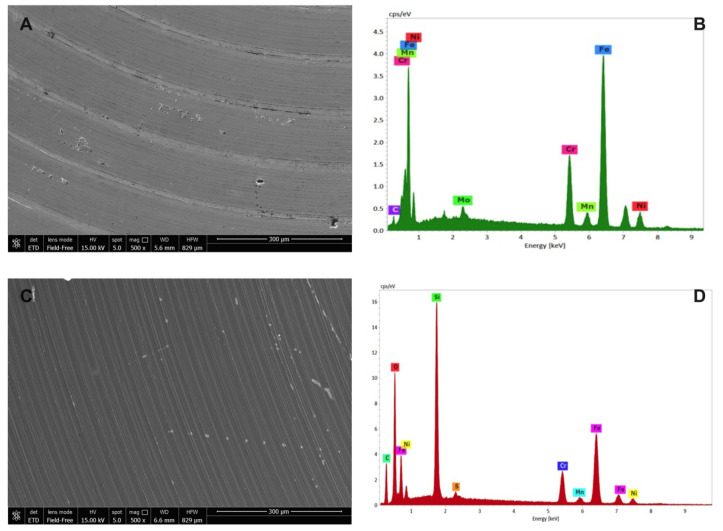
Environmental scanning electron microscope morphological analysis on (**A**) uncoated and (**C**) SiO_x_C_y_H_z_-coated stainless-steel disks observed at 300 μm, along with their respective (**B**,**D**) X-EDS microanalysis.

ESEM images of the uncoated and SiO_x_C_y_H_z_-coated stainless-steel disks, along with their respective X-EDS microanalysis, are reported in Figure 6.

As expected, X-EDS microanalysis of uncoated stainless steel showed the presence of chromium, nickel and molybdenum, characteristic of the AISI 316L quality (Figure 6B), while the morphological picture revealed an inhomogeneous surface with the presence of some crevices and pores (Figure 6A). On the other hand, SiO_x_C_y_H_z_-coated stainless-steel disk surface appeared more homogeneous, indicating a uniform layer on the surface whose composition was confirmed by the X-EDS microanalysis, which showed the presence of SiO_x_C_y_H_z_ (Figure 6C,D).

### 2.4. Contact Angle Analysis and Surface Energy Calculation

The surface wettability, energy, and component contribution (polar and nonpolar) of the analyzed samples are reported in Table 1, showing results of the steady-state contact angle (CA) measurements for all the surfaces wetted with polar (water) and nonpolar (diiodomethane) liquid. The standard deviation was computed considering three repeated tests conducted on each surface.

In general, CA depends on chemical composition and surface roughness. In fact, the wider the CA is, the lower the surface tension is, and the more water-repellent the surface proves to be. 

The increased hydrophobicity of specimens came from the dual effect of (1) nonpolar chemical groups of the coating, which reduced the surface energy of samples, and (2) surface roughness, which enhanced the water repellency according to the Wenzel model [26].

**Figure 7:**
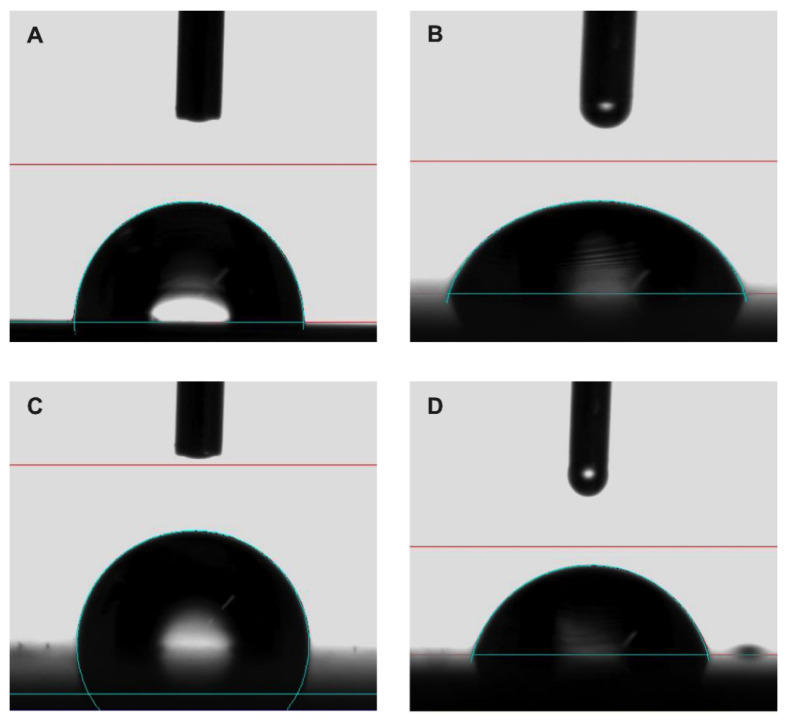
Representative contact angle images of (**A**,**C**) water and (**B**,**D**) diiodomethane on uncoated and coated sample, respectively.

In this case, the introduction of nonpolar chemical groups of the coating had a stronger effect than roughness: the polar component contribution decreased from an average value of 4.43 to 0 mJ m^−2^ after coating, while such a value did not change with surface roughness of uncoated disks. As a consequence, uncoated samples showed a low CA (average value 90.95°), while coated ones showed a wider CA (average value 113.55°), with the expected increase in hydrophobicity. The images of CA of water and diiodomethane drops on uncoated and coated surfaces are shown in Figure 7.

## 3. Discussion

In this study, we confirmed the antibacterial effect of an FCM-certified amorphous SiO_x_C_y_H_z_ coating deposited on stainless-steel disks with three different roughness (R0.25, R0.5, and R1 μm), and provided new insights on the time required to achieve the decrease in bacterial counts.

Indeed, in a previous study, we used the same SiO_x_C_y_H_z_-coated stainless-steel surfaces with three different roughness, the same starting inoculum (10^6^ CFU/mL), the same bacterial strains, and set the observation time after 12 h [5]. A five-logarithm decrease was observed without any sanitizing treatment both against Gram-positive and Gram-negative bacteria, while their complete removal was reached only after UVC and alcohol application.

Surprisingly, in this study, except for some samples (*Escherichia coli* at R0.5 and 0.25, *Salmonella Typhimurium* at R0.25, *Yersinia enterocolitica* at R1 and 0.25, *Staphylococcus aureus* at R0.25, *Listeria monocytogenes* at R1, 0.5, and 0.25, *Bacillus cereus* at R0.25, and *Enterococcus faecalis* at R0.5 and 0.25), a five-logarithm decrease was visible after 1 h on coated surfaces without any sanitizing treatment and inoculated [27] with Gram-negative and Gram-positive bacteria. As for the aforementioned strains, the time required to achieve the same decrease ranged between 30 min and 6 h.

Conversely, a complete bacterial removal was observed after 30 s^−1^ min application of alcohol and already after 15 s under UVC irradiation against both bacterial groups.

If, on one hand, we did not report a direct correlation between surface roughness of SiO_x_C_y_H_z_-coated samples and the antibacterial effect, on the other hand, CA observations demonstrated an increased hydrophobicity caused by the interaction of nonpolar chemical groups, which reduced the surface energy of treated samples. It is noteworthy that CA values of all coated surfaces are more than 90° range, and the polar components are zero, thus indicating a not wettable surface, which therefore resulted as more prone to preventing bacterial adhesion.

The antibiofouling activity is, therefore, of fundamental importance since, indirectly, the coating could also prevent biofilm formation and significantly reduce the use of corrosive sanitizing treatments.

According to such a claim, our results are in agreement with those achieved by Epstein et al., who first evaluated the use of slippery liquid-infused porous surfaces with antibiofouling activity as potential antimicrobial and antibiofilm solutions in the clinical, industrial, and consumer environment [28]. The authors firstly reported a 99.6%, 97.2%, and 96% bacterial attachment reduction over a 7-day period against *Pseudomonas aeruginosa*, *Staphylococcus aureus,* and *Escherichia coli*, respectively, under both static and physiologically realistic flow conditions. The surprisingly long-lasting period of efficacy was in contrast with other nanostructured superhydrophobic surfaces on which a biofilm formation was observed within hours.

Similarly, a 7-day period was also investigated to assess the possible microbiocidal activity of copper surfaces coated with silver nanoparticle cluster coatings against *Escherichia coli* O157:H7 and *Candida auris* [29]. Bacterial and fungal suspensions were challenged with coated surfaces and a significant reduction in their viability was observed, ~90% of *Escherichia coli* O157:H7 and ~100% *Candida auris*, respectively, thus representing more evidence of durable antibacterial surfaces.

Silver and copper, but also magnesium, nanoparticle-based bactericidal coatings were also successfully employed by Benetti et al. against *Escherichia coli* ATCC 25922 and *Staphylococcus* aureus ATCC 6538 strains, thus confirming the pivotal role of the coating’s physicochemical properties in tailoring the response against such pathogens [30].

It is also worth noting that many other literature reports shed light on different and equally reliable nanotechnological approaches to modify food contact surfaces (gold wrinkles [31], flexible hierarchical wraps [32], and a covalently tethered and flexible perfluorocarbon layer [33]) to prevent or reduce bacterial transmission and biofilm formation [34]; however, most of these were questioned for the possible migration of material from the surface to the food.

In fact, the low cytotoxicity of the SiO_x_C_y_H_z_ coating, which was within the range posed by regulations (EC) No. 1935/2004 [2], No. 1881/2006 [35], and No. 450/2009 [36], confirmed that the coating prevented metal ion release and that the antibacterial effect was rather ascribable to a lack of adherence of bacterial strains, possibly classifying the coated surface also as antibiofouling [37]. In fact, antibiofouling surfaces are known to prevent or disadvantage microorganism attachment due to topographical or chemical modifications [38].

Furthermore, as confirmed by ESEM images, SiO_x_C_y_H_z_ coating could have improved surface microtopography by completely covering crevices, pores, and other defects, also decreasing the number of attachment and proliferation sites for microorganisms that, on the contrary, were clearly visible on uncoated surfaces.

## 4. Materials and Methods

### 4.1. Samples and Coating 

**Figure 8:**
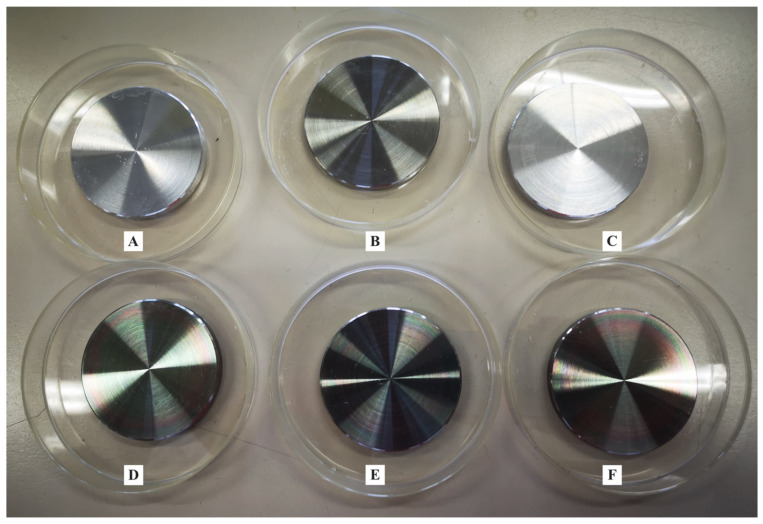
Representative image of stainless-steel disks of different R_a_ (**A**–**C**) uncoated and (**D**–**F**) SiO_x_C_y_H_z_-coated.

Three hundred and eighty-four round-shaped stainless-steel (AISI 316L, compliant with EN 10204 3.1) disks with a 5 cm diameter, 0.5 cm thickness, and three different surface roughness (R_a_) 0.25 ± 0.02 μm (R0.25, *n* = 128), 0.5 ± 0.03 μm (R0.5, *n* = 128), and 1 ± 0.06 μm (R1, *n* = 128) were used to carry out the experiments. One hundred and ninety-two disks were coated with a SiO_x_C_y_H_z_ coating approved for food contact, according to FCM certification [5,39] (Figure 8D–F), while the remaining 192 disks were left uncoated (Figure 8A–C).

ESEM images of the uncoated and SiO_x_C_y_H_z_-coated stainless-steel disks, along with their respective X-EDS microanalysis, are reported in Figure 8.

### 4.2. Contact Angle Analysis and Surface Energy Calculation According to ASTM D7490

Contact angle was observed using an optical contact angle apparatus (OCA 15 Plus, Data Physics Instruments GmbH, Filderstadt, Germany) equipped with a high-resolution CCD camera and a high-performance digitizing adapter and measured using an SCA20 software (Data Physics Instruments GmbH). Tested disks (one coated and one uncoated) were fixed and kept flat throughout the analysis by means of a special sample holder with parallel clamping jaws.

The static contact angle of water in air (*θ*, °) was measured by the sessile drop method, by gently dropping a droplet of 4.0 ± 0.5 μL of Milli-Q water (18.3 MΩ cm) onto the substrate, according to the so-called pick-up procedure: a droplet hanging down from the needle is laid on a solid surface by raising the sample stage until solid/liquid contact is made, at 23 ± 1 °C and 50 ± 2% relative humidity (RH).

The static contact angle was measured as the angle between the baseline of the drop and the tangent at the drop boundary. From the contact angle of a polar and a nonpolar liquid (water and diiodomethane, respectively) it is possible to calculate the surface free energy (mJ m^−2^) of the solid surfaces using the Owens, Wendt, Rabel, and Kaelble (OWRK) method [40].

### 4.3. Microbiological Analysis

American Type Culture Collection (ATCC) Gram-negative (*Escherichia coli* ATCC 25922, *Salmonella Typhimurium* ATCC 1402, *Yersinia enterocolitica* ATCC 9610, and *Pseudomonas aeruginosa* ATCC 27588) and Gram-positive (*Staphylococcus aureus* ATCC 6538, *Enterococcus faecalis* ATCC 29212, *Bacillus cereus* ATCC 14579, and *Listeria monocytogenes* NCTT 10888) strains were assayed in this study. All strains were grown in Tryptic Soy Broth (TSB, bioMérieux, Florence, Italy), incubated at 37 °C for 24 h, and activated by two successive transfers.

### 4.4. Inoculum Preparation

One hundred μL of the overnight cultures of each strain were transferred to 10 mL TSB and incubated at 37 °C with shaking. Cultures were spectrophotometrically measured at 600 nm after 5 h, and the viable cell count was determined by plating onto Tryptic Soy Agar (TSA). According to the procedure previously described [5], approximately 10^6^ CFU/mL of each bacterium was inoculated onto each stainless disk.

### 4.5. Time-course Assay, Sanitizing Procedures, and Surface Swabbing

For each bacterial strain, eighteen coated and eighteen uncoated disks (three for each roughness) underwent two different sanitizing treatments, i.e., UV (UVC, 253 nm, *n* = 9) and alcohol 70% (*n* = 9), while 12 (one for each roughness and treatment, both coated and uncoated) were used as controls.

A sterile swabbing was carried out at different times (0, 15″, 30″, 1′, 5′, 15′, 30′, 1, 2, 4, and 6 h) by surface friction. Then, the tip of the swab was placed in a test tube with 1 mL of 0.9% saline and vortexed for one minute. Serial tenfold dilutions of resuspensions were spread onto appropriate agar plates for the viable cell count. The colonies were counted following incubation at 37 °C for 24 h.

### 4.6. Cell Culture

Mammal fibroblasts NCTC clone 929 (L cell, L-929, derivative of Strain L) (ATCC^®^ CCL-1^TM^), were purchased from LGC Standards S.r.L. (Milan, Italy), grown in Dulbecco’s Modified Eagle’s Medium (DMEM) supplemented with 10% fetal bovine serum (FBS), 100 g/mL streptomycin, 100 U/mL penicillin, and 2 mM glutamine (Euroclone Spa, Milan, Italy), and cultured in a humidified incubator at 37 °C with 5% CO_2_.

### 4.7. Neutral Red Assay

Neutral red assay (TOX4 kit, Merck Life Science S.r.L., Milan, Italy) was used to assess the cytotoxicity of SiO_x_C_y_H_z_-coated stainless-steel samples with a direct extraction according to the ISO 10993–5:2009, Annex A. Vehicle (supplemented culture medium), test sample (extract derived from SiOxCyHz coated stainless-steel disk immersion in the vehicle), negative sample (extract derived from High Density Polyethylene USP Reference Standard immersion in the vehicle), and positive control (extract derived from latex immersion in the vehicle) were tested on a 24-well plate containing a subconfluent cell monolayer, subdivided as described in Table 2.

Test sample and negative control were immerged in the extraction vehicle in order to reach a weight/volume ratio of 3 cm^2^/mL for 24 h at a temperature of 37 °C in dynamic conditions. Conversely, positive control was prepared in order to reach a weight/volume ratio of 6 cm^2^/mL.

From subconfluent culture (80% of confluency), 0.5 mL of cell suspension was pipetted to the 24-well plate. The plate was incubated at 37 °C in a 5% CO_2_ atmosphere, allowing cell sedimentation and the constitution of a subconfluent monolayer. After 24 h, and verification that a subconfluent monolayer was present, the supernatant was removed and replaced with 0.5 mL of extract. The plate was incubated in a thermostat at 37 °C in a 5% CO_2_ atmosphere for 24 h.

This procedure was repeated for positive and negative controls. After this contact time, the plate was observed under an inverted microscope (Eclipse TE2000, Nikon, Japan), and biological reaction was evaluated according to a 0 to 4 scale as follows: 0 = discrete intracytoplasmic granules, no cell lysis, no reduction of cell growth; 1 = no more than 20% of the cells are round, loosely attached and without intracytoplasmic granules (or show changes in morphology), occasional lysed cells are present, only slight growth inhibition observable; 2 = no more than 50% of the cells are round, devoid of intracytoplasmic granules, no extensive cell lysis, no more than 50% growth inhibition observable; 3 = no more than 70% of cell layers contain rounded cells (or are lysed), cell layers not completely destroyed, more than 50% growth inhibition observable; 4 = nearly complete or complete destruction of cell layers.

Each well was treated with 1 mL of neutral red (NR) medium for 3 h. Thereafter, each well was washed with Dulbecco’s phosphate-buffered saline, totally dried, and then treated with 1 mL of NR desorb solution and placed on a stirrer for 10 min to homogenize the solution. Spectrophotometric analyses of wells were performed by an HP 8452 diode array spectrophotometer (GMI Inc., St. Paul, MN, USA) at 540 nm.

### 4.8. Environmental Scanning Microscopy Analysis (ESEM)

Scanning electron micrographs were obtained using a Nova Nano SEM 450 (Thermo Fisher Scientific, Waltham, MA, USA) in low-vacuum mode, with an energy-dispersive X-ray microanalysis system (X-EDS, QUANTAX-200, Bruker Nano Analytics, Berlin, Germany) using computer-controlled software. Each sample was mounted on an aluminum stub via double-sided adhesive tape and observed without sputtering at low vacuum (100 Pascal), with an accelerating voltage of 15 kV, working distance 5.6–6.6 mm, and standard acquisition resolution 1536 × 1024.

X-EDS microanalyses (133 eV resolution, amplification time 100 μs, and measuring time 60 s) for spectra were performed at 500–2000× original magnification. Areas of ~30 μm × 30 μm were selected for images at 500× original magnifications, while areas of ~2 μm × 2 μm at 2000× original magnifications were investigated.

### 4.9. Statistical Analysis

Data were analyzed using GraphPad Prism 8 software (GraphPad Software Inc., La Jolla, CA, USA). All data are presented as means ± standard deviation and were first checked for normality using the D’Agostino–Pearson normality test. Differences between cell viability of samples were analyzed using the Kruskal–Wallis test with Dunn’s multiple comparisons test. * *p* < 0.05 was considered significant.

## 5. Conclusions

We can conclude that amorphous SiO_x_C_y_H_z_ coating (Nanoxham-D^®^) induced a five-logarithm bacterial load decrease in a time frame of 1 to 6 h if not sanitized, achieving a complete bacterial load removal within a maximum of 1 min if sanitized with alcohol, and within 15 s if sanitized with UVC. Due to the lack of any cytotoxic effect, the coating can also be endowed with antibiofouling activity and, therefore, considered safer and not in need of any toxic, polluting, and corrosive sanitizing procedure.

Moreover, the deposition of amorphous SiO_x_C_y_H_z_ coating on stainless-steel disks changed the wetting behaviors of treated samples, with contact angles increasing from 90.25° to 113.55°, realizing a transformation from hydrophilicity to hydrophobicity and without polar components. This transformation has tremendous potential in various technological applications, including the food industry.

The lack of a biofilm formation assessment within the evaluation period, as well as the observation of the extent of bacterial persistence on the SiO_x_C_y_H_z_-coated surface, may represent limitations of this study. It is therefore our intention to deepen both aspects to better address the problem of surface contamination and cleaning, in particular in the food industry, by tuning the best combination of sanitizing treatment and the surface coating required to achieve the best and most long-lasting results in terms of bacterial attachment and proliferation inhibition.

## Figures and Tables

**Table 1:** Contact angle, surface energy, and component contribution of uncoated and coated samples.

Sample	*θ, °*		Surface Energy (mJ m^−2^)	Components (mJ m^−2^)	
	*θ_water_*	*θ_diiodomethane_*		Polar	Nonpolar
Uncoated (0.25 μm)	90.25 ± 5.24	75.41 ± 1.62	23.52	4.39	19.27
Coated (0.25 μm)	112.84 ± 7.65	72.92 ± 1.15	20.74	0	20.85
Uncoated (0.5 μm)	91.28 ± 5.33	76.45 ± 1.74	23.79	4.44	19.35
Coated (0.5 μm)	113.73 ± 7.87	73.61 ± 1.28	20.88	0	20.88
Uncoated (1 μm)	91.33 ± 5.41	76.24 ± 1.63	23.59	4.45	19.41
Coated (1 μm)	113.55 ± 7.71	73.82 ± 1.30	20.91	0	20.89

**Table 2:** Schematic representation of the Neutral red assay on a 24-well plate according to the ISO 10993–5:2009, Annex A.

Vehicle	Vehicle	Vehicle	Vehicle	Vehicle	Vehicle
Negative control	Negative control	Negative control	Negative control	Negative control	Negative control
Positive control	Positive control	Positive control	Positive control	Positive control	Positive control
SiO_x_C_y_H_z_ coating	SiO_x_C_y_H_z_ coating	SiO_x_C_y_H_z_ coating	SiO_x_C_y_H_z_ coating	SiO_x_C_y_H_z_ coating	SiO_x_C_y_H_z_ coating

## Data Availability

The data presented in this study are available on request from the corresponding authors.
